# An apprenticeship model in the training of psychotherapy students. Study protocol for a randomized controlled trial and qualitative investigation

**DOI:** 10.1371/journal.pone.0272164

**Published:** 2022-08-23

**Authors:** Heidi Brattland, Katrine Høyer Holgersen, Patrick A. Vogel, Timothy Anderson, Truls Ryum

**Affiliations:** 1 Nidelv Community Mental Health Center, St. Olav’s University Hospital, Trondheim, Norway; 2 Department of Psychology, the Norwegian University of Science and Technology (NTNU), Trondheim, Norway; 3 Department of Psychology, Ohio University, Athens, Ohio, United States of America; Flinders University, AUSTRALIA

## Abstract

**Background:**

One approach towards advancing the quality of mental health care is to improve psychotherapists’ skills through education and training. Recently, psychotherapy training has benefitted from adapting training methods from other professions (e.g., deliberate practice). The apprenticeship model has a long history in skill trades and medicine, but has yet to be adopted in training mental health professionals. This study aims to investigate the impact of apprenticeship training on clinical psychology students’ skills.

**Methods:**

In a pragmatic mixed-methods trial, 120 first year students in a Master’s degree clinical psychology program will be randomized to either training-as-usual or training-as-usual plus psychotherapy apprenticeship. In the intervention group, students will participate, over a period of 10 weeks, in weekly treatment sessions together with licensed therapists at outpatient mental health and substance use treatment clinics. Outcomes are assessed post-intervention and at two-year follow-up. The main outcome measure is the Facilitative Interpersonal Skills (FIS) performance test. Additional self-report measures tap self-efficacy, self-compassion, worry, rumination, and stress. Weekly reflection log entries written by the students will be qualitatively analyzed in order to gain an in-depth understanding of the learning process. Students’ and therapists’ experiences with the intervention will be explored in focus group interviews.

**Discussion:**

To the best of our knowledge, this is the first controlled study to investigate the impact of apprenticeship as an isolated training component in the education of clinical psychologists. The study is designed so as to yield a comprehensive understanding of an approach which could prove to be a valuable supplement to the existing educational methods in this field and ultimately, contribute to improve the quality of mental health care.

## Introduction

Psychotherapists vary considerably in their abilities to help their patients [1, 2]. As argued by Callahan and Watkins Jr [3, p. 211], high-quality psychotherapist training is “perhaps the most impactful systems-level intervention available to our field”: Any single therapist will treat thousands of patients in the course of their career and the effective improvement of each patient’s mental health yields multiplicative societal effects. Applying a training principle common in other educational fields such as medicine [4], but largely overlooked in the training of clinical psychologists, this study protocol describes an exploratory randomized controlled trial (RCT) and qualitative study into the impact of apprenticeship training for clinical psychology students.

The education of psychotherapists typically consists of a combination of didactic methods (e.g., coursework, reading treatment manuals, discussions of cases and treatment approaches) and clinical practice under supervision. Does training-as-usual facilitate the development of therapist expertise, defined, as recommended by Tracey and colleagues [5], as a demonstrable and progressive improvement of performance and/or client outcomes?

Regarding didactic methods, the overall conclusion from several literature reviews [6–9] is that while activities such as coursework and reading might increase students’ perceived and declarative knowledge, they are not alone sufficient to change their behaviors or improve client outcomes. Likewise, supervision may be more facilitative of declarative knowledge than of practical skills. Consistent with this notion, a recent review and meta-analysis of supervision research [10] reported consistently small effects of supervision on client-rated treatment outcomes [see also 11, 12]. It is likely that the addition of experiential and interactive methods is necessary for declarative knowledge to translate into improved performance, i.e. procedural knowledge. Perhaps not surprisingly, therapists typically describe practical experience working with clients as essential to their professional development [e.g., 13]. However a series of investigations of large naturalistic data sets found little or no evidence of an improvement of client outcomes with increasing clinical experience for trainees [14–17] or licensed therapists [18]. This body of research indicates that the combination of didactic methods and increased clinical experience under supervision might be less conductive to the progressive improvement of trainees’ patient outcomes than their subjective experience might perhaps suggest.

Beyond didactic methods and supervision, a new development in psychotherapist training is the application of training methods found to characterize expert performers in other fields, such as sports or music. Deliberate practice (DP) [19] involves practicing isolated skills or behaviors repeatedly outside of the performance situation. In the context of psychotherapy training, this involves, for instance, performing role plays where the trainee practices isolated skills (e.g., expressing empathy) or specific psychotherapy techniques. For further suggestions of how these principles can be applied to psychotherapy training, see for instance Miller and colleagues, [20], Rousmaniere and colleagues [21] and the Theravue electronic platform, www.theravue.com. The notion that DP might help improve trainees’ therapy skills has received some experimental support [22–27] but the evidence for an impact of DP on client outcomes is, to date, indirect [e.g., 28]. Indeed, meta-analyzing DP in diverse fields, Macnamara and colleagues [29, 30] found that DP had a limited influence on performance in low-predictability environments, that is, performance settings in which the range of possible actions is high and circumstances can change while the performer is planning and executing an action–such as is the case in psychotherapy.

In sum, evidence does not unequivocally support the combination of didactic methods and practice seeing patients under supervision with which most therapists are trained. While the application of DP techniques gives cause for optimism, their feasibility and impact on treatment outcomes in the low-predictable context of psychotherapy remain to be demonstrated. The exploration of supplementary training methods is warranted, and perhaps especially experiential methods that address the complexity of psychotherapy as it is practiced in real-world treatment settings.

Performing a task together with someone who knows it better is, arguably, a basic mechanism for human knowledge and skill transmission. We propose that the benefits of training-as-usual might be augmented by a simplified, time-limited psychotherapy apprenticeship intervention in which students of psychotherapy regularly sit in on therapy sessions conducted by licensed therapists. Apprenticeship training addresses aspects of a craft which cannot be learned by verbal communication or in simulated settings alone. As defined by social anthropologist Gowlland [31, p. 760],

apprenticeship can be characterized as a mostly non-didactic way of teaching and learning, grounded in a local context and dependent on participation of the learning in work related-activities; the acquisition of skills during an apprenticeship involves, among others, social participation and interaction, observation and imitation, and engagement through the senses with tools and context.

Drawing upon Albert Bandura’s theoretical framework of self-efficacy [32, 33], we expect psychotherapy apprenticeships to increase students’ beliefs in their ability to perform psychotherapy successfully in the future. According to this theory, knowing how to do a task (i.e., declarative knowledge) is not all it takes to perform well at it. Individuals with higher self-efficacy perform better because their positive self-appraisals and expectations help them approach tasks in a flexible manner, utilize adaptive coping mechanisms, and persist when faced with obstacles.

Bandura [34] theorized that one’s self-efficacy in a given area is influenced by four sources: *Mastery experiences* of being able to succeed in performing behaviors or tasks in that area, *vicarious experiences* of watching social role models similar to oneself perform such behaviors or tasks successfully, *verbal persuasion* in the form of positive feedback and encouragement from others, and one’s own level of *emotional arousal* while performing the task. Psychotherapy apprenticeships might work to improve psychotherapy students’ self-efficacy through all four sources. Most notably, the exposure to relatable therapist role models performing their craft in a real-life setting is likely to be a powerful vicarious experience. Moreover, in therapy sessions students can perform simple psychotherapy tasks (e.g., expressing warmth and positive regard for the patient, sharing their understanding of what the patient is experiencing, history-taking), thereby gaining mastery experiences. Any encouragement or positive feedback that therapists give to students following such experiences is a source of verbal persuasion that is directly linked that performance. Finally, repeated exposures to real-life therapy in the presence of a more experienced other may habituate psychotherapy students to the emotional challenges involved in working with individuals in distress, thereby decreasing their arousal and increasing their self-efficacy. We find this aspect particularly important in light of psychotherapy students’ high degree of worry about their abilities to perform therapy [35, 36] and novice therapists’ elevated anxiety levels in treatment sessions [13, 37], which is likely to draw their attention away from the unfolding interaction with the patient [35].

From the experienced therapists’ perspective, apprenticeship offers a way to transmit experience-based knowledge to a new generation of practitioners. It also provides an extra set of eyes and ears in the therapy room. Our own experience working with students is that their “naïve stance” can provide new, and sometimes surprising, perspectives on our own work. Moreover, we find students’ positive energy and enthusiasm for our craft refreshing and energizing.

Our literature search identified few attempts to apply the principle of apprenticeship to psychotherapy training, and then only as one of several components in comprehensive training programs. Examples include the Cognitive Apprenticeship Model of psychotherapy training and supervision [38, 39] and the Progress Cascading Model [40]. In a non-controlled study, the latter model was applied to the training in exposure therapy for obsessive-compulsive disorder [41]. Here, an improvement in trainees’ therapy delivery relative to before training was demonstrated. To the best of our knowledge however, apprenticeship has never been tested as an isolated component and in a controlled design in the context of psychotherapist training.

The main objective of the current randomized trial and qualitative study is to explore the potential benefits of supplementing the existing curriculum with a psychotherapy apprenticeship for first-year Master’s program students in a clinical psychology program. In the intervention group students will observe or co-treat patients together with licensed therapists at outpatient mental health and substance abuse treatment clinics. As a proxy to client outcome data, the main outcome will be Facilitative Interpersonal Skills (FIS); a series of studies [42–45] have demonstrated that this practical skills test is a meaningful indicator of overall therapist effectiveness. Additional quantitative outcomes as well as qualitative data from both students and therapists will be collected to yield a deeper understanding of the impact of apprenticeship experiences.

The following research questions will be investigated:

1. Does the addition of apprenticeship to training-as-usual increase psychology students’ Facilitative Interpersonal Skills (FIS) more than training-as-usual alone?
2. Is the hypothesized impact of apprenticeship on FIS associated with an increase in self-efficacy and a decrease in stress over the course of the practicum?
3. Is apprenticeship training beneficial to students’ levels of worry, rumination, and self-compassion?
4. Are students’ FIS scores associated to their personality traits, interpersonal functioning, social skills, attachment security, capacities for mentalization, and the group climate of their training-as-usual practicum course, and do these variables moderate the hypothesized impact of apprenticeship on FIS?
5. Are the hypothesized gains obtained through apprenticeship training maintained over a two-year period?
6. What are the salient aspects of therapy sessions for first-year students, and what characterizes the learning process in apprenticeships?
7. How does working together with student apprentices impact licensed therapists, and is apprenticeship a feasible training model in mental health care?

## Materials and methods

The protocol is reported according to the SPIRIT guidelines [46] and a SPIRIT checklist can be found in the Supporting Information, S1 Table.

### Study design

**Fig 1:**
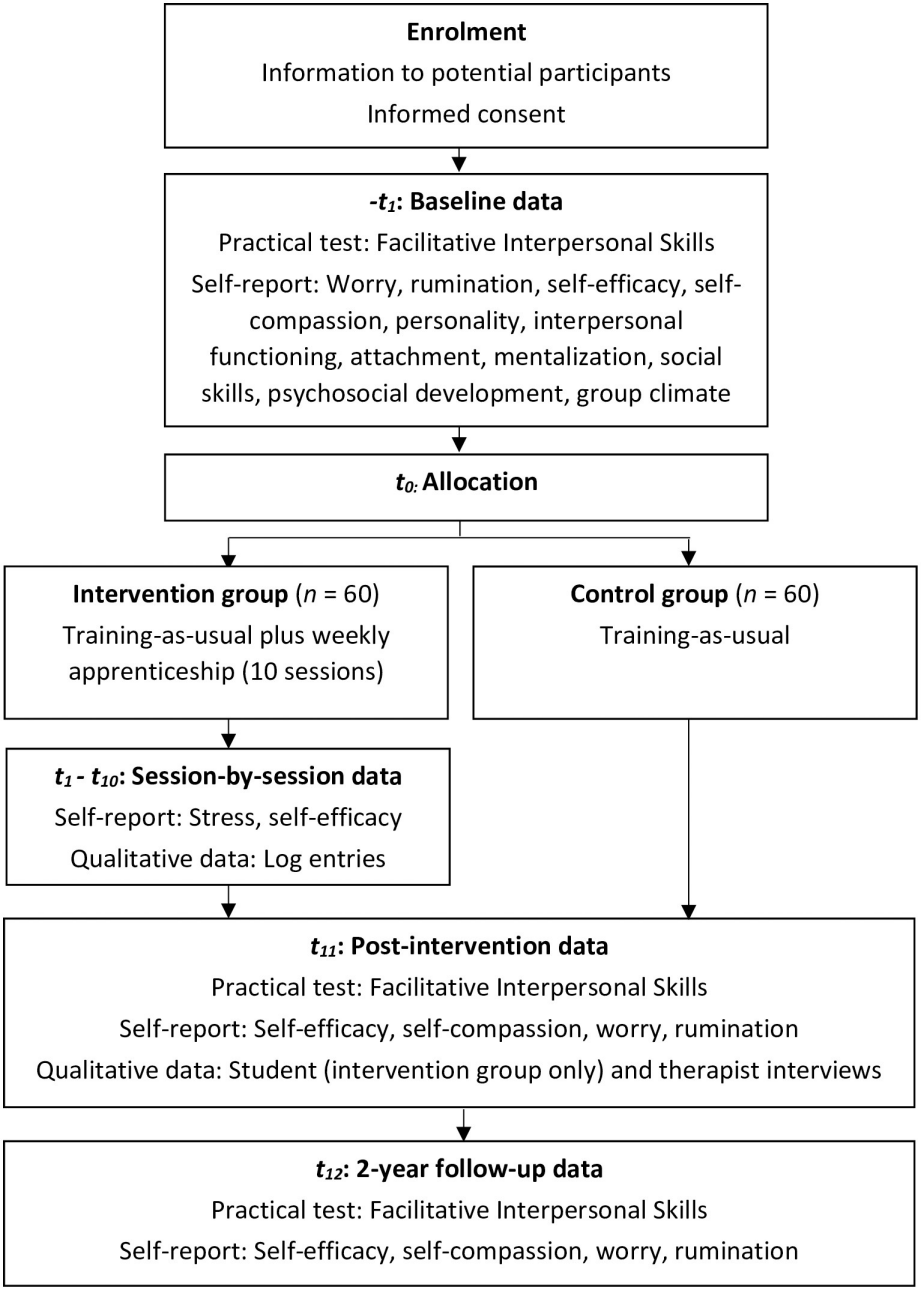
Flow chart with an overview of the study design.

A pragmatic mixed-methods explorative study, consisting of (1) a randomized controlled, parallel group, two-arm, superiority trial comparing training-as-usual to training-as-usual plus apprenticeship, and (2) a qualitative investigation of students’ and therapists’ experiences of the apprenticeship intervention. The study design is illustrated in Fig 1.

### Study setting

Participants will be recruited from the clinical psychology program at the Norwegian University of Science and Technology (NTNU) in Trondheim, Norway. A clinical psychologist license in Norway is earned through a six-year master’s level clinical psychology track. The first three years of the study program are mainly dedicated to the acquisition of theoretical knowledge on psychology, psychopathology, and psychotherapy, with limited and only indirect practical experience with psychotherapeutic work. From year four onwards, students have internship practicums with child and adult patients followed by a full-time, six-month externship practicum.

The apprenticeship training will take place at three clinics at the St. Olavs University Hospital, Norway, two of which are community health centers and one, a substance use treatment clinic. As part of the specialized mental health care system, these treatment facilities serve a population of adult individuals (18 years of age or older) with intermediate to severe mental health or substance use disorders. In their apprenticeships, students will rotate between different outpatient teams. While some of these teams are general psychiatric teams (i.e., treat mental health problems of all diagnostic categories) that cover different geographical areas, others are dedicated to specific patient populations such as patients with eating disorders, young adults or individuals experiencing mental health crises, or to specific tasks such as the early assessment of newly referred individuals for whom the suitability for specialized mental health care is unclear.

### Participants and eligibility criteria

Student participants will be first-year students at the clinical psychology program (*N* = 120), randomized 1:1 to training-as-usual or training-as-usual plus apprenticeship. All first-year students are eligible to participate in this study (no exclusion criteria). The apprenticeship intervention will be performed by therapist employees (licensed clinical psychologists, psychiatrists, psychiatric nurses, or from other professions) of the three participating clinics. All therapist employed outpatient teams in these clinics are eligible to participate.

### Interventions

#### Training-as-usual (control group)

The apprenticeship intervention will be evaluated against the standard education for psychotherapists in Norway. At the NTNU, subjects covered during the first year of the clinical psychology program are the history and methodology of psychology, mental health disorders, and introduction to cognitive, developmental, and personality psychology. In addition to these theoretical subjects, students attend a clinical practicum course with the aim of gaining a basic understanding of clinical psychological work and in particular, the importance of relational competency and communicative skills for the practice of psychotherapy. This course is workshop-based and conducted in groups of approximately 10 students, one instructor, and one student assistant. The main teaching methods are lectures, exercises and discussions. As part of this course, all first-year students attend a one-day observational practice with a clinical psychologist.

#### Apprenticeship training (intervention group)

In addition to training-as-usual, students will attend weekly treatment sessions with licensed therapists at the mental health and substance use clinics over a period of 10 weeks, each week with a different therapist and patient. A set weekday and time of day for each clinic will be determined for the apprenticeship intervention, and therapist participants will make appointments with patients they view as suitable at that time. Students will rotate between different therapists so as to gain the broadest possible experience base, effectively meeting a new therapist and patient for each apprenticeship session.

In sessions, students will either observe passively or assume the more active role of a co-therapist. Participating therapists are encouraged to include the student in sessions as much as they see fit. Respecting individual preferences and the organic nature of therapeutic conversations, the study protocol does not dictate the degree of student in-session participation but instead, leaves the decision up to each particular therapist, student, and patient in that specific session. Students will likely be increasingly able over their apprenticeship period to participate actively in sessions, especially if paired with therapists who are comfortable working with co-therapists and with patients who draw students into the conversation. Because of the rotation of students between therapists, variations in degree of participation will largely be due to chance and the students’ own preferences.

Following each treatment session students will have a 15–20 minutes reflective conversation with the therapist. This discussion will be unstructured so as to allow for any questions or topics that the student might find important or interesting. The learning process will then be further facilitated by the writing of reflective log entries focusing on the students’ experiences of participating in each treatment session. Members of the research team meet with student participants before and after each apprenticeship training session in order to administer outcome measures and continually monitor the students’ adherence to the protocol. Therapists will be invited to a meeting with the research group during the intervention period to exchange experiences and practical tips regarding their work with the students, and facilitate their adherence to the protocol.

The apprenticeship intervention is based on results from a pilot trial (unpublished) in which quantitative and qualitative data from both therapists and students were collected, analyzed, and used to refine the intervention and study procedure.

### Measures and outcomes

See Table 1 for an overview of all measures and outcomes.

**Table 1:** Schedule of enrolment, interventions, and assessments.

	STUDY PERIOD													
	Enrolment	Allocation	Post-allocation										Close-out	
TIMEPOINT	*-t*_*1*_	*t*_*0*_	*t*_*1*_	*t*_*2*_	*t*_*3*_	*t*_*4*_	*t*_*5*_	*t*_*6*_	*t*_*7*_	*t*_*8*_	*t*_*9*_	*t*_*10*_	*t*_*11*_	*t*_*12*_
**ENROLMENT**:														
**Informed consent**	x													
**Randomization**		x												
**INTERVENTIONS**:														
***Training-as-usual (control)***														
***Apprenticeship (intervention)***			x	x	x	x	x	x	x	x	x	x		
**ASSESSMENTS**:														
***Baseline variables***:														
***NEO-FFI***	x													
***IIP-64***	x													
***ECR-N12***	x													
***MentS***	x													
***MPD***	x													
***SSI***	x													
***GCQ***	x													
***Outcome variables***:														
***FIS***	x												x	x
***Stress scale***	x												x	x
***PSWQ***	x												x	x
***RRS***	x												x	x
***CASES***	x												x	x
***FSCRS***	x												x	x
Stress scale*			x	x	x	x	x	x	x	x	x	x		
Self-efficacy*			x	x	x	x	x	x	x	x	x	x		
***Qualitative data***:														
Log entries*			x	x	x	x	x	x	x	x	x	x		
Student interviews*													x	
***Therapist interviews***													x	

FIS, Facilitative Interpersonal Skills; PSWQ, Penn State Worry Questionnaire; RRS, Rumination Response Scale; NEO-FFI; FSCRS, Forms of Self-Criticising/Attacking & Self-Reassuring Scale; CASES, Counselor Activity Self-Efficacy Scale; IIP64, Inventory of Interpersonal Problems; ECR-N12, Experiences in Close Relationships; MentS, Mentalization Scale; SSI, Social Skills Inventory; MPD, Measures of Psychosocial Development; GCQ, Group Climate Questionnaire. Asterisks indicate that data are collected in the intervention group only.

#### Primary outcome

The main outcome change from baseline in the Facilitative Interpersonal Skills (FIS) test [43], performed at baseline, post-intervention and two-year follow-up. It is a practical skills test in which a test person responds to video stimuli of actors portraying clients in various difficult or challenging moments in therapy. The situations ***are*** designed to represent behavior in the various areas of the Interpersonal Circumplex model [47], such as an angry and confrontational client or a quiet and withdrawn client. The test person is instructed to respond verbally to each particular situation, as if they themselves were that client’s therapist, in a way they believe might be helpful. Responses are video recorded and subsequently rated in accordance with the FIS manual [48] for the degree of interpersonal skills on a scale from 1 (*not characteristic*) to 5 (*extremely characteristic*). The FIS assesses eight interpersonal skills, namely 1. Verbal Fluency, 2. Hope and Positive Expectations, 3. Persuasiveness, 4. Emotional Expression, 5. Warmth, Acceptance, and Understanding, 6. Empathy, 7. Alliance Bond Capacity, and 8. Alliance Rupture-Repair Responsiveness. The overall mean score will be utilized in analyses.

The original FIS stimuli clips were translated into Norwegian language and re-recorded with student actors by authors of this study protocol. A preliminary validation study [49] found interrater agreement and internal consistency on the Norwegian FIS test to be excellent. In this trial, students in both conditions will be tested on six clips each at pre- and post-interventions and at two-year follow-up. At each measurement occasion clips are selected to represent a balanced and varied range of challenging clinical situations. Responses will be rated by psychology students trained in the FIS methodology and blind to treatment condition and time of assessment.

#### Secondary outcomes

All secondary outcomes are pen-and-paper self-report and administered at baseline, post-intervention, and at two-year follow-up. For all measures, the main parameter of interest is change from baseline in overall mean score.

Self-efficacy will be assessed with an adaptation of the Counselor Activity Self-Efficacy Scales, CASES [50] to suit first-year students with limited experience and knowledge of psychotherapy. CASES is a 59-item measure of self-perceived capability to perform basic helping skills, manage session tasks, and negotiate challenging counseling situations and challenging issues. Items are scored on a 10-point Likert scale from 0 (*no confidence*) to 9 (*complete confidence*). The scale has been shown to be highly reliable and sensitive to change [50]. For the purpose of this study we selected 11 items that we thought our participants would be able to respond to, and rephrased the instructions from “*Indicate how confident you are in your ability to use each of the following helping skills effectively*, *over the next week*, *in counseling most clients*” to “*In an imagined therapy situation*, *how confident are you in your ability to…*”. One item was selected from the basic helping skills dimension (i.e., “*capture and understand the messages that clients communicate*”), four from the session management dimension (e.g., “*help your client to understand his or her thoughts*, *feelings*, *and actions*”), and six from the challenging client issues dimension (e.g., “*client is extremely nervous*”).

Students’ degree of worry will be assessed with the Penn State Worry Questionnaire, PSWQ [51]. A total of 16 items are scored on a 5-point Likert scale, from 1 (*not at all typical*) to 5 (*very typical*). A sample item is “*I know I shouldn’t worry about things*, *but I just can’t help it*”. The measure is widely used and has been shown to possess good psychometric properties and to tap a construct independent of other indicators of anxiety or depression [51].

The Rumination Response Scale, RRS [52], will measure rumination as a method of coping with negative mood. A total of 22 items are scored on a 4-point Likert scale, from 1 (*almost never*) to 4 (*almost always*). A sample items is “*go away by yourself and think about why you feel this way*”. The scale has been demonstrated to be reliable and valid, and to measure trait-like coping styles that are not confounded by state effects of depressed mood [53, 54].

Self-compassion will be assessed with the Forms of Self-Criticising/Attacking & Self-Reassuring Scale, FSCRS [55], a 22-item measure of self-criticism and self-reassurance. Items are scored on a 5-point Likert scale from 0 (*not at all like me*) to 4 (*extremely like me*). A sample item is “*I find it easy to forgive myself*”. An analysis of data from 12 individual studies utilizing this instrument [56] found it to be robust and reliable in both clinical and non-clinical samples, with established normative data for each population.

#### Additional measures

Participants in the intervention group will be administered the following self-report measures at each apprenticeship occasion in order to examine changes in self-efficacy and stress over the course of the apprenticeship: A generic one-item, 10-point Likert scale of subjective stress immediately before attending treatment sessions (Instruction: “*Notice how you’re feeling right now*. *Indicate on the scale below how much stress you’re experiencing in this moment*”); four items from the session management dimension of the Counselor Activity Self-Efficacy Scales, CASES [50] (see description of the measure above), and a generic one-item, 10-point Likert scale of confidence in the therapist role (Instruction: “*Indicate the alternative that you feel represents you best right now in your development as a therapist*: *How confident do you feel in the role as a therapist*?”).

At baseline, a battery of questionnaires will be administered to participants in both conditions in order to examine predictors of FIS across conditions and potential moderators to the impact of the apprenticeship training. Personality will be assessed with the NEO Five-Factor Inventory, NEO-FFI [57], a 60-item measure of the five basic personality factors Neuroticism, Extraversion, Openness, Agreeableness, and Conscientiousness. Items are scored on a 5-point Likert scale, from 1 (*strongly disagree*) to 5 (*strongly* agree). The NEO-FFI is a reliable, valid, and widely used personality measure [e.g., 58].

Self-assessed social skills will be assessed with the Social Skills Inventory, SSI [59], a 90-item measure of three types of verbal and non-verbal communication skills: expressivity, sensitivity, and control over communication. Items are scored on a 5-point Likert scale from 1 (*not at all like me*) to 5 (*exactly like me*). A sample item is “*I am often told that I am a sensitive*, *understanding person*”. The instrument has been reported to have good psychometric properties including test-retest reliability and convergent and discriminant validity [60].

Interpersonal functioning will be assessed with the Inventory of Interpersonal Problems, IIP-64 [47, 61], a 64-item measure of maladaptive relationship behavior. Items are scored on a 5-point Likert scale, from 0 (*not at all*) to 4 (*extremely*). Eight subscales correspond to octants in the Interpersonal Circumplex (domineering/controlling, vindictive/self-centered, cold/distant, socially inhibited, non-assertive, overly accommodating, self-sacrificing, and intrusive/needy). The inventory is widely used both in treatment and research and its validity and reliability are well documented [e.g., 62, 63].

Adult attachment quality will be assessed with the Experiences in Close Relationships–Short form, ECR-S [64]. A total of 12 items are scored on a 7-point Likert scale, from 1 (*disagree strongly*) to 7 (*agree strongly*). A sample item is “*I find it difficult to allow myself to depend on romantic partners*”. Like the original, 36-item version of ECR, the psychometric properties of ECR-S have been shown to be sound [64].

Capacity for mentalization will be assessed with the Mentalization Scale, MentS [65]. A total of 24 items are scored on a 5-point Likert scale, from 1 (*completely incorrect*) to 5 (*completely correct*). Three dimensions are assessed: self-related mentalization, other-related mentalization, and motivation to mentalize. A sample item is “*Often I cannot explain*, *even to myself*, *why I did something*”. The MentS is a relatively new inventory whose consistency, reliability, and validity has been reported to be adequate to good [65, 66].

Psychosocial functioning will be assessed with the Identity and Intimacy subscales (28 items) of the Measures of Psychosocial Development MPD [67]. The full, 112-item instrument assesses the positive and negative ends of each of Erikson’s eight psychosocial stages as well as the resolution of these stages. Items are scored on a 5-point Likert scale from 1 (*not at all like* me) to 5 (*very much like* me). Adequate to strong construct validity, test-retest and internal consistency was reported in the MPD manual [67].

The quality of the group climate in students’ practicum groups, which all students attend as part of training-as-usual, will be assessed with the Group Climate Questionnaire—Short form, GCQ [68]. A total of 12 items are scored on a 7-point Likert scale, from 1 (*not at all*) to 7 (*extremely*). Three subscales are assessed: Engagement, Avoidance, and Conflict. The GCQ is the most frequently used group process measure in the group psychotherapy literature and satisfactory reliability of the measure has been reported in several studies [e.g., 69, 70].

To examine and control for the potential impact of stress/heightened activation on performance on the FIS, students will be asked to complete a generic one-item measure of subjective stress (similar to the one administered at each apprenticeship occasion, see Secondary outcomes), immediately before the FIS test. Following the FIS test participants are asked to rate, on 10-point Likert scales, how much they feel that what they said would be helpful to the patient and to what degree they feel their responses represent their skills at a therapist, considering their current educational stage.

#### Qualitative data

Written reflective logs will be completed by students in the intervention group immediately following each treatment session they attend. The logs serve the dual purpose of facilitating the learning process by encouraging students to reflect upon salient aspects of their apprenticeship experience, and of providing a session-by-session insight into the learning process. In the logs, students will be asked to describe and reflect upon their immediate experiences of participating in each particular treatment session. Students in the intervention group as well as therapist participants will be invited to participate in focus group interviews focusing on the overall experience and impact of the apprenticeship training. Each focus group will consist of four to seven students or therapists and a moderator. All interviews will be audio-recorded and transcribed verbatim.

### Procedures

#### Enrolment

Prospective student participants will be informed about the study and encouraged to volunteer in lectures at the university. Written, informed consent will be required for student participants and will be obtained by members of the research team prior to randomization. Therapist participants will be recruited by presenting the study to each of the various outpatient teams at the three clinics.

To facilitate recruitment, student participants in both conditions will be compensated with bus passes for the duration of the apprenticeship intervention. Control condition participants will be offered a workshop on interpersonal skills following their completion of the post-intervention measures. No data will be collected from this workshop; instead, it is intended as a compensation for the students’ participation.

#### Allocation

Following informed consent and baseline measures, participants will be randomized 1:1 to the experimental or control condition. The randomization will be conducted by one of the members of the research team using a web-based randomization program for medical research (https://webcrf.medisin.ntnu.no). Participants will be informed immediately about what condition they are assigned to. Blinding of participants or researchers will not be practically feasible.

#### Assessment

Baseline data (-*t*_1_) will be collected prior to randomization and consists of the FIS skills test, administered by members of the research team, as well as several self-report measures of secondary outcomes and potential correlates of the FIS. See above for details about measures and outcomes. Session-by-session self-reported stress- and self-efficacy measures as well as reflective log entries will be completed by students in the intervention group at each clinic and immediately before and after each treatment session they attend (*t*1 –*t*_10_). Following the 10-week apprenticeship period (*t*_11_), students in both conditions will complete the FIS-test at the university, using different stimulus clips from those that were used pre-intervention, as well as self-report measures of secondary outcomes. At this point in time, students in the intervention group as well as therapists will be invited to focus group interviews. Follow-up data (*t*_12_) will be collected from students in both conditions two years after the completion of the apprenticeship condition, and consist of the FIS test as well as self-reported secondary outcomes.

#### Data management

Participants will be given a numeric study ID which is identifiable by a coding list only accessible to the principal investigator. Data will be manually double-entered by research assistants who are blind to allocation.

### Sample size

Sample size was determined based on parameters on the main outcome measure FIS from three samples [42, 43, 71]. A sample size of 100 yields an 80% probability for detecting a medium intervention effect (*d* = 0.5) with a one-sided alpha level of .05. Allowing for 20% attrition, we aim to include 120 student participants.

### Statistics and data analyses

Quantitative outcomes will be analyzed using regression models in which post-intervention and follow-up scores will be predicted by condition, controlled for pre-intervention scores and other possible covariates. Longitudinal models will examine the development over time on the session-by-session stress and self-efficacy measures. Missing data will be handled with multiple implementation. Qualitative data will be thematically analyzed.

### Data monitoring

There are no anticipated harmful consequences of participation in this study and therefore, no stopping guidelines are developed and no interim analyses are planned.

### Ethics approval and consent to participate

The study will be conducted in accordance with the Declaration of Helsinki. It was approved by the Regional Ethics Committee in Mid-Norway (REK) as an extension of a larger research project (2012/1498). Participation is voluntary and written, informed consent is obtained. No patient data will be collected in the project; in interviews and written log entries, students and therapists will be instructed not to share any identifiable client information. Students in the intervention group will sign a confidentiality agreement prior to the apprenticeship intervention. Therapists will obtain verbal consent to have a student co-therapist present in sessions from those of their patients that they consider to be suitable. The therapists will be responsible for the treatment delivered.

### Status and timeline

This publication is based on protocol version 1 (05-04-2021). Recruitment for the trial began on 09-20-2021, and is expected to be completed by October 2023.

## Discussion

This randomized controlled trial and qualitative investigation will explore the potential impact of applying an educational method widely practiced in other fields, the apprenticeship, to the training of future psychotherapists. We consider it to be a promising supplementary element to the traditional (e.g., coursework, clinical practice under supervision) as well as the newer (e.g., deliberate practice) methods of psychotherapist training. In their apprenticeships, students will be exposed to the complexity—and sometimes messiness and confusion—of psychotherapy as it is practiced in actual clinical settings. A variety of ways to respond to challenging interactions in therapy will be demonstrated by relatable role models. Importantly, students will have the opportunity to discuss and gain insight into what considerations lie behind these role models’ actions with their clients, as well as to try out their own therapeutic skills under close guidance. We expect this to be facilitative of students’ interpersonal skills, to increase their self-efficacy, decrease their performance anxiety and stress, and to advance their understanding of the practical psychological work. This would demonstrate a proof-of-concept by this study. We also expect it to be engaging and helpful for the experienced therapists to welcome student co-therapists into their therapy room.

### Strengths and limitations

The study has several important strengths. The experimental intervention is apprenticeship in a very simple form. The randomized controlled design will allow us to examine any additive effects of apprenticeship as a single element to training-as-usual while decreasing the influence of systematic biases on results. The qualitative data from several sources will help provide a more nuanced understanding of both students’ and therapists’ apprenticeship experiences. Interviews and reflective logs will supplement the quantitative measures, allow for unexpected findings to emerge, and be vital in generating hypotheses for future studies. We consider a sample of first-year psychology students to be ideal in testing the impact of apprenticeship on interpersonal skills; they are untainted by previous training and may have fewer preconceptions and be less preoccupied with the technical aspects of the specific treatment models than more advanced students perhaps would. Possibly, this demonstration and practical experience in real-world psychotherapy at the very beginning of their careers will provide them with a strong starting point from which to absorb and assimilate all subsequent learning experiences more effectively. The main outcome measure, the FIS performance test, assesses skills in standardized situations in which client variability is controlled for, and has been demonstrated to be highly predictive of client outcomes [72]. It is designed to tap therapist behaviors that are likely to be of importance regardless of what specific treatment model therapists adhere to and as such, we expect results to be generalizable to therapists of all theoretical orientations.

The main limitation of apprenticeship as a training method is perhaps the unpredictability of clinical work. In this study, there will be no control over what clients the students meet or what happens in sessions with those clients. Students’ exposure to challenging interpersonal interactions will be completely at random. It is also very possible that the therapists’ demonstration of interpersonal skills will be less than optimal. Arguably however, examples of what not to do can also be very educational. Moreover, observing therapists performing less than optimally can be a healthy antidote for students who may have perfectionistic tendencies. It is our hope that this early exposure to the complexity of psychotherapy, in conjunction with other educational methods, will help prepare them to the unpredictability that characterizes real-life clinical work [73].

Some of the design choices we have made inevitably result in certain limitations. The rotation of students between different therapists is intended to counteract therapist effects and ensure that students are exposed to a variety of different clients, therapists, and ways of conducting therapy. Disadvantages of therapist rotation is that students will be less able to form a secure relationship to one role model or to follow the full course of treatment with one client. Moreover, the apprenticeship intervention is shorter in duration and less intensive than the full-time apprenticeships that are perhaps more commonly practiced in other fields. The rationale behind this is both feasibility–the intervention needs to fit into the ongoing study program as well as the day-to-day practice at busy treatment clinics–and the acknowledgement of the importance of the other educational methods that this intervention is meant to supplement, but not replace.

### Implications

The aim of this study is to gain preliminary knowledge on a relatively simple and, hopefully, feasible training method intended to improve the treatment of mental health problems through the improved education of future therapists. Given the high prevalence and costs of mental health problems for individuals and society at large, we find the endeavor to educate more efficient psychotherapists of utmost importance.

### Dissemination plans

Upon completion, results will be published in peer-reviewed journals and presented at research conferences and other relevant arenas for research dissemination. Authorship to all future publications will be granted in accordance with the Vancouver recommendations. Any modifications to this protocol will be reported in subsequent publications.

## Supporting information

S1 Table SPIRIT checklist. (DOCX)
